# Influence of health behaviours on the incidence of infection and allergy in adolescents: the AFINOS cross-sectional study

**DOI:** 10.1186/1471-2458-14-19

**Published:** 2014-01-09

**Authors:** Esther Nova, David Martínez-Gómez, Sonia Gómez-Martínez, Ana M Veses, Maria E Calle, Oscar L Veiga, Ascensión Marcos

**Affiliations:** 1 Immunonutrition Group, Institute of Food Science and Technology and Nutrition. ICTAN-CSIC, C/Jose Antonio Novais 10, 28040 Madrid, Spain; 2 Department of Physical Education, Sport and Human Movement. Faculty of Teaching Training and Education, Universidad Autónoma de Madrid, Madrid, Spain; 3 Department of Preventive Medicine and Public Health, Faculty of Medicine, Universidad Complutense de Madrid, Madrid, Spain

**Keywords:** Allergy, Healthy habits, Infection, Overweight, Obesity, Sleep, IgE, Leptin

## Abstract

**Background:**

Some health behaviours are liable to affect the incidence of allergies and/or common infections in young people; however, the extent and ways in which these might occur are mostly unknown. This study examines the association of health behaviours related to physical activity, sedentariness, diet and sleep with allergy and infection symptoms in adolescents, and also with biological markers that might mediate disease incidence.

**Methods:**

The study comprised a total of 2054 adolescents (50.7% girls) from the Madrid region of Spain. The incidence of infection and allergy symptoms three months prior to the study was obtained from a self-administered questionnaire. Physical and sedentary activities, height and weight, food habits and sleep duration were also self-reported and their influence on infection and allergy incidence was assessed by logistic regression analysis. Blood biomarkers (IgE, eosinophil percentage, leptin, interleukin (IL)-2, IL-4, IL-5 and IL-10) were evaluated in a subsample of 198 subjects.

**Results:**

Adequate sleep duration (OR = 0.79, 95%CI: 0.64 to 0.97) and unhealthy weight status (overweight/obesity) (OR = 1.35, 95%CI: 1.04-1.74) were independently associated with decreased and increased allergy incidence, respectively. No significant association was observed with infection incidence. IgE and leptin differed between adolescents with and without allergy symptoms. In regression models IgE was significantly associated with inadequate sleep duration and leptin with weight status.

**Conclusion:**

Excess weight and inadequate sleep duration are independently associated with the incidence of allergy symptoms in adolescents. Adequate sleep duration and weight during adolescence might be relevant for a decreased risk of suffering allergy symptoms.

## Background

The most frequent infectious diseases as well as the highly prevalent allergic diseases are a considerable burden to the Health Care system [1,2]. Prevention of both disease states would certainly benefit from a better understanding of the influence of relevant lifestyle factors and certain behaviours. Some factors related to the Western lifestyle have been linked to the higher prevalence of allergies and immune dysregulation through the “Hygiene hypothesis” and the broader “Wrong Life Style hypothesis” [3,4]. A general decrease in exposure to non-pathogenic micro-organisms is believed to impact the Th1/Th2 balance and promote immune responses favouring sensitization and allergy to common, non-harmful antigens.

Recent epidemiologic studies have demonstrated that the prevalence of allergies [1] and obesity [5] are both increasing concomitantly, suggesting that these diseases may be causally related [6], or perhaps share common risk factors such as sedentariness, inactivity [7], unhealthy food choices [8] or insufficient sleep duration [9]. The importance of maintaining a normal body weight seems also relevant with regard to the defence mechanisms against common infections [10]. All the mentioned factors are highly variable among adolescents. On the other hand, interactions among all these influencing factors might also occur, making it difficult to reveal their true impact on disease development.

All these bases considered, we aimed to analyse how different lifestyle factors related to physical and sedentary activities, sleep and dietary habits could influence the incidence rates of infection and allergy symptoms in Spanish adolescents, and secondly, to look into some blood biomarkers of allergy and inflammation that might be mediating such influence.

## Methods

### Study design and participants

Participants in the present study were those enrolled in the AFINOS (La Actividad Física como Agente Preventivo del Desarrollo de Sobrepeso, Obesidad, Alergias, Infecciones y Factores de Riesgo Cardiovascular en Adolescentes/Physical Activity as a Preventive Measure Against Overweight, Obesity, Infections, Allergies and Cardiovascular Disease Risk Factors in Adolescents) cross-sectional study between 2007 and 2008. The AFINOS study design and protocols have been described in detail elsewhere [11].

In brief, the AFINOS study was designed to assess health status and lifestyle factors through a survey completed by a representative sample of adolescents, aged 13 to 17 years, from the Madrid region of Spain. Secondary schools were randomly selected according to the geographic distributions of adolescents in this region. The sample size was calculated taking 0.05 as the maximum permissible error (reliability of 95%) and based on an estimated prevalence of overweight and obese adolescents of 20% [5]. The final sample size calculated at 1998 adolescents was increased by 20% to compensate from possible dropouts or data losses to give a final sample size of 2400 participants of both sexes. Of these subjects, 2054 (1012 boys and 1042 girls) providing valid data on the incidence of allergies and infections were included in the current study. This study was conducted according to the guidelines laid down in the Declaration of Helsinki (as revised in Hong Kong in 1989 and in Edinburgh, Scotland, in 2000), and all procedures involving human subjects were approved by the Ethics Committee of the Puerta de Hierro Hospital (Madrid, Spain) and the Bioethics Committee of the Spanish National Research Council. Written informed consent was obtained from all adolescents and their parents/guardians.

### Infection and allergy frequency

Suffering from allergy symptoms (itchy eyes and nose, watery eyes, sneezes and runny nose, skin rash, asthma or respiratory trouble without fever) and suffering from infection symptoms (common respiratory illness with breathing trouble, cough, expectoration and fever and/or gastrointestinal infection with diarrhoea and abdominal pain plus fever) during the last three months, was self-reported using ad-hoc questions included in the epidemiological questionnaire. This was fulfilled from September 2007 to April 2008, which covers the period of common winter infections and part of seasonal allergies (i.e. cupressaceae family).

### Health behaviours

Overall physical activity was assessed using the validated Spanish version of PACE + (Physician-based Assessment and Counselling for Exercise) questionnaire for adolescents [12]. This questionnaire uses 2 questions to assess physical activity: Q1: “Over the past 7-d, on how many days were you physically active for a total of at least 60 min per day? Q2: “Over a typical or usual week, on how many days are you physically active for a total of at least 60 min per day?” Both questions have a scale of 0 to 7 days. Adolescents were classified into two groups (active/inactive) according to the PACE + criterion that considers physically active adolescents those who engage in physical activity at least 5 days/week using the mean of both questions [13].

Adolescents were also asked to self-report how much time they usually spent watching television, and using computer/video games for leisure activities and doing homework both at schooldays and at weekends (a weighed mean was calculated). Sleep duration at night (hours) on usual schooldays and weekends was also reported. For these behaviours, participants were classified into two groups (compliant and non-compliant) as follows: watching television less than 2 hours/day [14], doing homework at least 1 hour/day, playing with play station/computer less than 1 hour/day and at least 8 hours/day sleep at night [15]. Adolescents’ dietary habits regarding recommended fruit intake (at least 2 servings a day) and daily early-morning breakfast consumption (yes/no) were also assessed from the responses given in a self-reported eating habits and food frequency questionnaire included in the survey [16]. Adolescents were asked to self-report their body weight and height. However, these anthropometrical measurements were taken by standard procedures in the subsample of the population that underwent blood measurements. Body mass index was calculated as: weight/height^2^ (kg/m^2^). Overweight and obesity prevalence was calculated using the BMI age- and sex-specific cut offs proposed by Cole et al. [17]. Z-score of BMI was calculated with the data obtained from the Spanish growth charts and tables by Sobradillo et al. [18].

### Biological variables

IgE, eosinophil counts, leptin and several serum cytokine levels were evaluated for their putative implication as mediating factors in the associations of health behaviours and disease. These biological variables were selected from the set of biological variables measured in a subsample of 198 adolescents of the AFINOS study, which has been described somewhere else [11]. Briefly, eosinophil percentage was assessed using an automated cell counter (ABX 120DX Horiba, Spain), IgE was quantified using a chemoluminescence method (ADVIA CENTAUR, Siemens), and the cytokines interleukin (IL)-2, IL-4, IL-5 and IL-10 were quantified by high sensitivity multiple analyte immunologic technique (xMAP Techonology) using a commercial kit (Millipore Corp., Billerica, MA, USA) and the flow cytometry equipment Luminex-100IS (Luminex Corporation, Austin, TX, USA). Finally, leptin concentrations were obtained with the same technology and kit manufacturer (Millipore Corp.). The intra and inter-assay variation coefficients were always lower than 5% and 12%, respectively, for the analytes measured.

### Data analysis

All the variables are presented as mean (SD) and percentages. Differences between sexes were examined by one-way analysis of variance and Chi-squared test for continuous and categorical variables, respectively. Binary logistic regression analyses were applied to examine the associations between: 1) weight status and disease incidence (infection and allergy separately) and 2) seven other health behaviours and disease incidence (infection and allergy separately). Odds ratios and 95% confidence intervals were calculated for all associations using either a two or a three additive model. The first model showed crude odds ratio (OR) and 95% confidence interval (CI), whereas the second model showed multivariate-adjusted estimates controlling for age and sex. The third model showed multivariate-adjusted OR and 95% CI controlling for age, sex and BMI Z-score when appropriate. A fourth and final, fully adjusted model included age, sex and those health behaviours that had shown significant or borderline associations (P < 0.1) with disease incidence in the second or third model, in order to test if those associations are independent of each other. In the logistic regression models, the group not meeting the recommendation for each health behaviour was considered the reference group. Finally, binary logistic regression was performed to assess any association between health behaviours influencing the same disease, in order to further rule out confounding effects that might be underlying the relationships between health behaviours and a disease.

Biological variables that might have a role in the associations found in the above described analyses were studied. Differences in the mean values of these variables were assessed between adolescents reporting incidence and absence of disease symptoms (allergy and infection), respectively. A normal distribution of the variables IgE, leptin, and all the interleukins was obtained after their logarithmic transformation. The differences between groups were then assessed by Student’s *T* test. Biomarkers that differed depending on disease incidence were further analysed as surrogate markers of the disease. Thus, they were used as dependent variables in linear regression analyses that included as independent variables those health behaviour that had resulted associated with the disease incidence in the logistic regression analyses performed with the whole adolescent sample (n = 2054). This linear regression analyses were adjusted by age, sex and Z-score as appropriate. Analyses were performed using the SPSS for Windows statistical software package version 19 (SPSS Inc., Chicago, IL, USA) and the significance level was set at P < 0.05.

## Results

Descriptive characteristics are shown in Table 1. The incidence of infection and allergy during the three months previous to the questionnaire completion were higher in girls than in boys (P < 0.001). The percentage of girls meeting the recommendation “perform more than one hour of physical activity at least 5 days a week” was lower than in boys (P < 0.001). Girls, however, showed a significantly higher frequency of fruit consumption (P = 0.033). Differences among sexes were also observed for time spent using computer/video games and doing homework (both P < 0.001) but no differences appeared for time spent watching TV or sleep duration.

**Table 1:** Demographic and lifestyle characteristics of adolescents

	***All***	***Boys***	***Girls***	***P***
N	2054	1013	1041	
*Physical characteristics*				
Age, yr	14.8 ± 1.3	14.8 ± 1.3	14.8 ± 1.3	0.208
Weight, kg	58.4 ± 11.0	62.5 ± 11.9	54.3 ± 8.4	<0.001
Height, cm	166.5 ± 9.2	170.5 ± 9.6	162.7 ± 6.9	<0.001
Body mass index, kg/m^2^	21.0 ± 2.9	21.4 ± 3.2	20.5 ± 2.6	<0.001
Body mass index, Z-score	−0.12 ± 1.00	−0.06 ± 1.00	−0.18 ± 0.99	<0.010
*Infection and allergy frequency*				
Infection incidence in the last 3 mo., %	32	28	36	<0.001
Allergy incidence in the last 3 mo., %	36	28	44	<0.001
*Health behaviours*				
Overweight + obesity, %	17.5	24.8	10.5	<0.001
Physical activity ≥5 d/week, %	17	22	12	<0.001
TV viewing <2 hr/d, %	70	71	68	0.160
Sleep duration ≥8 hr/d, %	67	68	65	0.174
Fruit consumption ≥2 servings/d, %	22	20	24	0.033
Use of screen devices at leisure time <60 min/d, %	79	68	89	<0.001
Homework ≥60 min/d, %	64	55	72	<0.001

Physical characteristics are expressed as mean (SD).

Table 2 shows the influence of weight status on the incidence of infections and allergies. Excess weight was positively associated with the incidence of allergy (OR = 1.343, 95% CI 1.047, 1.723, P = 0.020, in the sex and age adjusted model), but showed no association with infections.

**Table 2:** Associations between weight status and incidence of infection and allergy in adolescents

	**Odds ratio (95% confidence interval)**		
	**Non-Overweight**	**Overweight/obese**	***P***
**Infection**			
(n)	1649	350	
Unadjusted	1 (Reference)	1.080 (0.842-1.384)	0.546
Sex and age adjusted	1 (Reference)	0.924 (0.714-1.194)	0.544
**Allergy**			
(n)	1644	351	
Unadjusted	1 (Reference)	1.091 (0.860-1.385)	0.473
Sex and age adjusted	1 (Reference)	1.343 (1.047-1.723)	*0.020*

The analysis of the influence of behavioural factors on the incidence of infection in the last three months showed no significant association between them (data not shown). A trend, however, was observed for a lower incidence of infection in those with a higher frequency of fruit consumption, which remained after adjusting by age and sex (OR = 0.796, 95% CI 0.631, 1.004; P = 0.054) and became significant in the model adjusted by age, sex and BMI Z-score (OR = 0.782, 95% CI 0.618, 0.989; P = 0.040).

**Figure 1:**
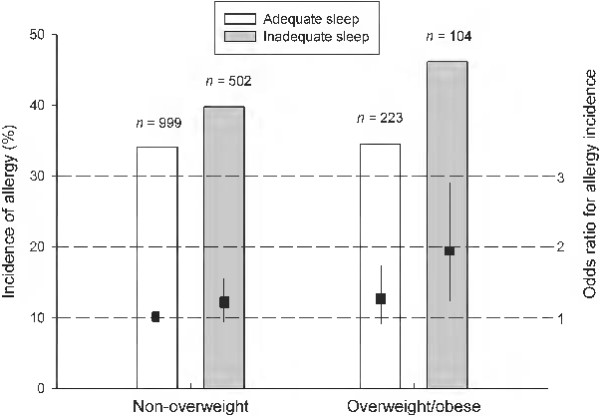
**Incidence of allergy in subsets of the adolescent population.** Incidence of allergy across sleeping habits and body weight categories among adolescents (n = 1828) aged 13–17 years, from Madrid, Spain. Simple boxes depict odds ratios and 95% confidence interval of allergy incidence for each category, in the sex and age adjusted model.

Among candidate health behaviours only adequate sleep duration showed a significant association with allergy incidence, both in the crude and adjusted models. Sleeping > or = 8 hours decreased the probability of allergy symptoms by 21% (OR = 0.786, 95% CI 0.638, 0.968; P = 0.024, in the age, sex and BMI Z-score adjusted model) (Table 3). A final fully adjusted model including both sleep duration and weight status, in addition to age and sex, was tested. This analysis showed that sleep duration (OR = 0.781, 95% CI 0.634, 0.963; P = 0.021) and weight status (OR = 1.346, 95% CI 1.039, 1.743; P = 0.024) were independently associated with allergy incidence. Hence, adolescents with inadequate sleep duration and suffering from overweight or obesity reported the highest frequency of allergies (Figure 1). The OR for each category is shown graphically in Figure 1. In order to rule out the influence of hidden confounding factors, we tested if sleep duration and overweight/obesity status were associated and the analysis showed no association between them. All analyses were repeated in boys and girls separately and in older and younger groups (13.00-15.49 and 15.50-17.99) and the results did not change substantially (data not shown).

**Table 3:** Associations between health behaviours and incidence of allergy in adolescents (n = 1794 to 2016)

		**Odds ratio (95% confidence interval)**		
	**n**	**Unadjusted**	**Sex and age adjusted**	**Sex, age and BMI Z-score adjusted**
**Physically active**				
< 5 days/week	1645	1 (Reference)	1 (Reference)	1 (Reference)
≥ 5 days/week	325	1.001 (0.781-1.282)	1.142 (0.886-1.472)	1.124 (0.868-1.455)
*P*		0.996	0.304	0.375
**Television viewing**				
≥ 2 hours/day	552	1 (Reference)	1 (Reference)	1 (Reference)
< 2 hours/day	1266	0.885 (0.721-1.788)	0.906 (0.735-1.116)	0.914 (0.739-1.130)
*P*		0.247	0.353	0.407
**School homework**				
< 1 hour/day	658	1 (Reference)	1 (Reference)	1 (Reference)
≥ 1 hour/day	1176	0.942 (0.773-1.147)	0.840 (0.686-1.029)	0.849 (0.690-1.043)
*P*		0.549	0.091	0.119
**Computer use and video games**				
> 1 hour/day	374	1 (Reference)	1 (Reference)	1 (Reference)
≤ 1 hour/day	1420	1.268 (0.995-1.616)	1.035 (0.803-1.334)	1.025 (0.793-1.326)
*P*		0.055	0.790	0.849
**Sleep duration**				
< 8 hours/day	622	1 (Reference)	1 (Reference)	1 (Reference)
≥ 8 hours/day	1254	0.743 (0.610-0.906)	0.771 (0.628-0.947)	0.786 (0.638-0.968)
*P*		*0.003*	*0.013*	*0.024*
**Breakfast**				
No	251	1 (Reference)	1 (Reference)	1 (Reference)
Yes	1722	1.184 (0.903-1.554)	1.025 (0.777-1.354)	0.997 (0.752-1.321)
*P*		0.222	0.860	0.981
**Fruit consumption**				
<2 servings/day	1567	1 (Reference)	1 (Reference)	1 (Reference)
≥2 servings/day	449	1.019 (0.820-1.267)	0.984 (0.789-1.228)	0.976 (0.780-1.223)
*P*		0.865	0.889	0.839

IgE, eosinophil percentage, serum cytokine and leptin concentrations were studied as potential biological factors with a role in the associations found between weight status and allergy incidence and between sleep recommendation compliance and allergy incidence. Only IgE and leptin showed significant differences between adolescents reporting presence and absence of allergy symptoms, respectively (P = 0.012 for IgE, and P = 0.017 for leptin) (Table 4); However, IgE was increased with allergy symptoms both, in boys and in girls, while leptin was only increased in girls with allergy symptoms but not in boys. Linear regression analyses were then performed with these biomarkers as dependent variables and including sleep recommendation and weight status as independent variables, adjusting the model for age and sex. The results showed that only meeting sleep recommendation (standardized beta [β] = −0.147, P = 0.037) and sex (β= − 0.187, P = 0.008) were significantly associated with IgE levels in this model. On the other hand, only weight status (β=0.389, P < 0.001) and sex (β=0.524, P < 0.001) were significantly associated with leptin in this model. The association of IgE and sleep duration, as well as that of leptin with weight status, were not qualitatively changed if groups defined by sex were analysed separately (data not shown).

**Table 4:** Biological variables in adolescents with and without allergy symptoms in the last three months

	**All (n = 198)**		**Boys (n = 101)**		**Girls (n = 97)**	
**Symptoms presence**	**No (n = 148)**	**Yes (n = 50)**	**No (n = 82)**	**Yes (n = 19)**	**No (n = 66)**	**Yes (n = 31)**
IgE (mg/dL)	127 ± 273	258 ± 483*	168 ± 319	321 ± 595*	75 ± 190	220 ± 405°
Leptin (pg/mL)	7043 ± 6196	10280 ± 8184*	4595 ± 4902	4602 ± 5682	10084 ± 6317	13761 ± 7555*
Eosinophils (%)	2.9 ± 2.8	3.0 ± 1.7	3.6 ± 3.6	4.1 ± 1.9	2.2 ± 1.0	2.4 ± 1.2
Interleukin-2 (pg/mL)	12.7 ± 22.3	15.7 ± 28.4	9.5 ± 13.1	18.5 ± 42.7	16.7 ± 29.6	14.1 ± 14.7
Interleukin-4 (pg/mL)	185 ± 280	170 ± 237	204 ± 305	208 ± 254	159 ± 243	141 ± 225
Interleukin -5 (pg/mL)	3.2 ± 21.8	1.6 ± 1.5	1.2 ± 1.5	1.4 ± 1.2	5.5 ± 32.5	1.6 ± 1.8
Interleukin −10 (pg/mL)	65 ± 181	50 ± 89	32 ± 33	46 ± 61	108 ± 264	52 ± 103

Values are mean ± SD.

*Significant differences between adolescents with allergy symptoms and without allergy symptoms. Student’s T test, P < 0.05; °P < 0.1.

## Discussion

This epidemiological study examined the association between several health behaviours and the incidence of allergy and infection symptoms reported in a self-administered questionnaire completed by 2054 adolescents. The results revealed that weight excess and shorter sleep duration were both independently associated with allergy incidence and no association was found between the overweight/obese status and sleeping less than eight hours in our population. In the literature, allergy has been associated with sleep disturbances in variable settings. Allergic rhinitis, for instance, is associated with sleep loss or an impaired sleep pattern [19] through nasal obstruction and the enlargement of tonsils and adenoids. On the other hand, allergy medication may impair sleep architecture for instance by increasing the latencies to sleep onset and reducing the REM sleep duration [20]. However, due to the cross-sectional nature of this study, we do not know the cause-effect direction of the association and it is not possible to rule out that lack of sufficient sleep is a risk factor for allergy onset instead of the more intuitive relationship between allergy nasal obstructive symptoms and sleep disturbances.

On the other hand, depression as comorbidity in allergic patients might explain why allergic people would sleep less hours. A published study by Williams et al. [21] found that 21% allergic patients showed brief recurrent depression and this disorder is associated with sleep disturbances. We analysed this relationship in the population of the current study and the OR for depression in those suffering allergy symptoms was 1.773 (95%IC: 0.944 – 3.330; p = 0.075).

Regarding the association found between overweight/obesity and allergy symptoms, previous epidemiological studies have also shown obesity to be related to allergy symptoms or to high serum IgE levels (a marker of atopy) [2,22-24]. However, others have not confirmed this association [25-27]. Furthermore, among studies finding a significant association, food allergy has been pinpointed as the driving force of the association in some studies [2,28] and rhino-conjunctivitis in another [25].

Regarding blood biomarkers in our study, we did not find that IgE was increased in adolescent with excess weight, but was associated with inadequate sleep duration, which supports the relationship found between shorter sleep and allergy incidence in the whole population. Published works have shown that sleep deprivation induces non-specific leukocyte activation and an increase in inflammatory and pro-inflammatory markers [29,30]. On the other hand, leptin, was only associated with weight status and thus remains a potential mediator of the association found between overweight/obesity and allergy incidence, although only in girls, since boys with allergy symptoms maintained normal leptin levels. In this regard, leptin and other fat derived inflammatory mediators have been proposed as a link between obesity and allergy [31,32].

We found no association between sleeping less than 8 hours per day and being overweight or obese. This is in contrast with a meta-analysis of epidemiological studies describing shorter sleep duration as an independent risk factor for higher body weight in children [33]. However, our finding is in agreement with a recent epidemiological study which found no independent association between insufficient sleep and childhood obesity [34].

Regarding food habits, we are not aware of any previous epidemiological study looking into the association between habitual breakfast consumption and daily fruit consumption and allergy incidence. However, some previous reports from epidemiological studies have referred decreased infection prevalence with increasing fruit consumption [35]. Noticeably, we found an almost significant value for this association in our population, which became significant in the model with additional adjustment by Z score.

To our knowledge this is also the first study assessing possible associations between habitual physical activity or sedentary habits and allergy incidence in adolescents. They were found to be unrelated. No associations were found either between these behaviours and infection incidence. A “J-shape” seems to describe the relationship between the amount of exercise and the infection risk in adults, with high intensity exercise increasing this risk. However, there are very few data on this relationship in children or adolescents. A recent review highlights the limited evidence, basically resulting from three studies, seemingly suggesting a protective effect of regular physical activity against respiratory infections in non-athletic children [36].

Finally, it is interesting to highlight the higher incidence of infection and allergy symptoms in the three months previous to the questionnaire completion in girls than in boys. Several reasons might explain these findings. Firstly, girls’ concern about their health seems to be higher [37] and induces a female to male dominance in self-reported allergic disease [38,39]. Secondly, several sex differences in allergic diseases have been identified in the literature, such as a higher prevalence of drug and/or antibiotic allergy in females [40,41].

This study has some limitations. Firstly, given the cross sectional nature of the assessments we cannot establish a cause-effect relationship of the associations, as we have already mentioned. Secondly, the assessment of allergy and infection incidence is based in self-reports and thus it is affected by the individual capacity to remember past events, which in addition is likely to be lower in the younger age adolescents. Thirdly, although several adjustments have been performed in the analysis, the influence of potential confounders not accounted for cannot be disregarded.

## Conclusions

This epidemiological study found that excess weight and inadequate sleep duration are independently associated with the incidence of allergy symptoms in adolescents. Although causality has not been established, maintaining healthy body weight and good restoring sleep during adolescence might be relevant guidelines when designing therapeutic options and preventive measures against allergy.

## Abbreviations

IgE: Immunoglobulin E; BMI: Body Mass Index; IL: Interleukin.

## Competing interests

The authors declare that they have no competing interests.

## Authors’ contributions

EN, DM-G, SG-M, MC, OLV and AM contributed to the design of the study. DM-G, SG-M, AV, and OLV performed the surveys and data collection. MC performed database supervision. EN, DM-G performed data analysis and interpretation of results. EN wrote the first draft of the manuscript. OLV and AM coordinated study procedures. All authors contributed to the writing of the manuscript’s final version. All authors read and approved the final manuscript.

## Pre-publication history

The pre-publication history for this paper can be accessed here:

http://www.biomedcentral.com/1471-2458/14/19/prepub
